# The analysis of the medical tourism expansion policy in Taiwan: a policy analysis using Kingdon’s multiple streams

**DOI:** 10.1186/s12961-024-01180-0

**Published:** 2024-08-14

**Authors:** Ying-Ju Yu, Nicole Huang, Hsu-Sung Kuo

**Affiliations:** 1 International Health Program, Institute of Public Health, College of Medicine, National Yang Ming Chiao Tung University, Taipei, Taiwan, ROC; 2 DreamWay International Healthcare Centre, Taipei Medical University Hospital, Taipei, Taiwan, ROC; 3 Institute of Hospital & Health Care Administration, National Yang Ming Chiao Tung University, Taipei, Taiwan, ROC

**Keywords:** Medical tourism, Free Economic Pilot Zones, Health policy, Taiwan, Kingdon’s multiple streams

## Abstract

**Background:**

Since 2006, Taiwan has actively pursued the development of its medical tourism industry. In 2013, the government sought to bolster this sector by integrating medical tourism into the Free Economic Pilot Zones. Despite narrowly missing the mark, the initiative failed to materialize into law. This qualitative study endeavors to discern the pertinent factors influencing the agenda-setting process for incorporating medical tourism into the Free Economic Pilot Zones in Taiwan.

**Methods:**

A comprehensive examination of policies concerning the legitimation of medical tourism within the Free Economic Pilot Zones was undertaken through semi-structured interviews and a thorough review of policy documents. Key informants were strategically selected using purposive and snowball sampling techniques. Thematic analysis was applied to scrutinize the amassed data and organize it within the framework of Kingdon's multiple streams.

**Results:**

In the problem stream, increasing financial strains and cost containment pressures under the National Health Insurance program have long driven health care providers to seek further opportunities in medical tourism. The existing barriers to expanding medical tourism in Taiwan included diplomatic tensions (specifically cross-strait relations), public concerns about commercialization of medical care and reduced their access to care, and legal and language barriers. Within the policy stream, factors such as franchise fees to support national health insurance, limited number of demonstration medical tourism sites and services allowed, the allowance of foreign medical personnel, regulations governing domestic physicians, the importance of demonstration, regulation, and accreditation, as well as restrictions on investment from China, were emphasized. The politics stream highlights factors such as governmental support, opposition from opposing parties, public concerns and critics from academia and non-governmental organizations, and skepticism from medical faculties.

**Conclusion:**

Acknowledging the recognized challenges in enacting the medical tourism provision of the Free Economic Pilot Zones Special Act and emphasizing the political will of leadership, a viable policy solution remained elusive. Although a window of opportunity existed for the passage of the bill, it waned as public concerns sidelined the issue from the national agenda. The Taiwan case underscores the necessity for meticulous consideration of issues, proposed solutions, and political dynamics to achieve successful policy enactment.

## Background

Medical tourism, which emerged in the late 1990s and has expanded explosively, with an anticipation to be grew at a compound annual growth rate (CAGR) of 11.59% from 2023 to 2032, is regarded as one of the major demonstrations of globalization globally [1–3]. Medical tourism, generally is described as people travelling across international borders from their country of residence to another country with the intention of accessing medical treatment [4–6], and may encompass the full range of medical services including urgent care, elective procedures, dental care, cosmetic surgery, health check-ups and fertility treatment [7–9]. The Asia Pacific region is projected to experience the most rapid growth in medical tourism in the upcoming years and is expected to lead the global market in the field [3, 10], of which Taiwan (Republic of China) is one of the top medical tourism destinations, along with India, Malaysia, Singapore, the Republic of Korea and Thailand [11].

Globally, several factors contribute to the rise of medical tourism. These include the high cost of medical care in developed countries, long waiting times for certain procedures, availability of advanced medical technologies and expertise in destination countries, and the appeal of combining medical treatment with tourism [5, 12–14]. Conversely, barriers such as concerns over quality and safety of medical procedures, regulatory, ethics and legal issues, cultural and language differences, lack of international supervision, and potential risks of post-operative complications without adequate follow-up care also significantly impact the industry [12, 13, 15–17].

Medical tourism is mainly offered by the clinics and hospitals at the destination countries, and has been introduced to the economic zones since 2002 in United Arab Emirates and has expanded to several other Asian countries, such as Japan, the Republic of Korea, Turkey and the People's Republic of China (PRC) [18–24]. Economic zones are mostly located within borders but outside customs boundaries, experiencing minimal government interference, with reduced or eliminated import taxes on raw materials and goods [25]. In 2013, the government of Taiwan sought to emulate other Asian countries by introducing medical tourism services into its economic zones, known as Free Economic Pilot Zones (FEPZs), with targets on both economic freedom and the development of the health industry.

The draft of “The Free Economic Pilot Zones Special Act” (FEPZs bill) was proposed by the Executive Yuan (the executive branch of Taiwan government, structured by the head of the premier and the cabinet members) in March 2013, and the development of FEPZs in Taiwan was carried out in two stages: in the first stage, the Executive Yuan approved the related regulations that did not require amendment of laws in August 2013; in the second stage, the bill needs to be enacted by the Legislation Yuan (Legislature, the Congress), and the FEPZs bill was submitted to Legislation Yuan in December 2013 [26]. The Legislative Yuan conducted a thorough review of the Act in April 2014; however, its passage has been pending due to ongoing disputes between the ruling and opposition parties [26, 27].

The FEPZs bill has eight chapters, including general provisions, application and management, treatment of foreigners and people from the PRC, taxation, standardization of untaxed goods and labor services, industrial activities, education and professional services, and penalty provision, aiming at developing intelligent logistics, international medical services (medical tourism), and value-added agriculture—all niche industries with high potential [28, 29]. The medical tourism regulations are listed in Articles 49 to 53 in Chapter 6 and are summarized in Table 1 [29].

**Table 1:** The Articles 49 to 53 in the FEPZs bill

Article	Descriptions
Article 49	The establishment of medical care corporations for medical tourism shall not be subject to the following restrictions: 1. Provisions of Article 49, Paragraph 1 of the Medical Care Act 2. Provisions of Article 50, Paragraphs 1 and 2 of the Medical Care Act The capital contributions of members, the number of directors, and the proportion of medical personnel and foreigners serving as directors among the members of the medical care corporations mentioned in the preceding paragraph shall be determined by the central health and welfare authority
Article 50	Medical tourism institutions may employ foreign medical personnel to practice within the institution, and the number or ratio of such foreign medical personnel employed shall be announced by the central health and welfare authority Foreign medical personnel mentioned in the preceding paragraph must be approved by the central health and welfare authority and are not subject to the requirement of holding a specialized professional certificate in the country. The qualifications, conditions, abolition and other management matters shall be determined by the central health and welfare authority Foreigners and foreign medical personnel covered by the preceding two paragraphs shall a not apply to residents of Hong Kong or Macao
Article 51	Domestic doctors who are not registered in medical tourism institutions are not allowed to perform medical services in medical tourism institutions However, this restriction does not apply to emergency situations, consultations, support between medical institutions, invited visits or with prior approval, and does not exceed the time limit stipulated by the central health and welfare authority
Article 52	Medical tourism institutions are not allowed to be contracted as healthcare service providers under the national health insurance
Article 53	Medical care corporations entitle operating medical tourism institutions shall pay an annual franchise fee to the central health and welfare authority based on a certain proportion of the total operating revenue from the previous year The commencement year for the payment of the franchise fee mentioned in the preceding paragraph, the percentage to be paid, the purpose and the ratio of each purpose shall be determined by the central health and welfare authority

Source: Ministry of Justice [79]. The original descriptions are in Mandarin and translated by the authors

According to the Taiwan Ministry of Health and Welfare (MOHW), the FEPZs allows the establishment of one or two demonstration sites within the FEPZs to offer medical tourism services [30]. Of these demonstration sites, only one hospital, with a maximum of 200 beds, is allowed. The services will mainly be limited to health checkups, cosmetic medicine, and routine surgery [30]. Medical administrators show a higher acceptance of the medical tourism provision of the FEPZs bill as the economic benefits are clearly lucrative [31, 32]. Despite the Taiwanese government’s emphasis on FEPZs representing a key initiative to create momentum in the economic growth of the country, the MOHW stated that legitimation of medical tourism in the FEPZs will absolutely not affect the medical interests of local citizens or the availability of the National Health Insurance (NHI) [33, 34]. Strong reactions from the public followed the approval of the first stage by the Executive Yuan in December 2013, leading to five public hearings held by the Legislative Yuan from March to April 2014 and an online forum conducted in May 2014 [35]. It was decided that, until the bill was passed in the Legislative Yuan, the second stage of the FEPZs program would not be initiated. Since then, one legislator proposed the bill in 2019, but the bill has not been listed on the agenda again [36].

Since policies play a fundamental role in health issues, it is crucial to identify and understand relevant factors that could impact the agenda-setting process.

Addressing this case is important for several reasons. Firstly, the FEPZs bill, proposed in 2013 and reviewed in 2014, has neither been passed nor rejected, indicating ongoing relevance and unresolved policy debates. Secondly, the bill's reintroduction in 2019 highlights its continued necessity and potential benefits for Taiwan's economic and healthcare landscape. Thirdly, there is a lack of research exploring government attitudes and support for medical tourism, as noted in the literature [37]. Lastly, cultural differences, such as uncertainty avoidance during the COVID-19 pandemic, have also significantly impacted medical tourism, emphasizing the need for a deeper understanding of various factors affecting this industry [38]. Therefore, examining this decade-long delay can contribute to academic and policy debates on effectively implementing such initiatives and fostering economic and healthcare advancements. However, this issue has not been explored by other scholars. This qualitative study aimed to better understand why the section of the FEPZs bill related to medical tourism was pending and the strategies and issues, including potential obstacles, during the legitimation of medical tourism into the FEPZs. To achieve this, Kingon’s multiple streams framework was suitable for the purpose and was utilized for this study.

## Conceptual framework: problem, policy and politics

John Kingdon’s multiple streams framework emerged in the mid-1980s [39] and is widely employed as a conceptual tool for comprehending the intricacies of policymaking, particularly in healthcare [40]. Kingdon’s theory builds upon the Garbage Can Model proposed by Michael D. Cohen, James G. March, and Johan P. Olsen [41]. While the Garbage Can Model focuses on organizational choices, Kingdon’s framework aims to understand how issues become prominent in policymaking [42]. According to Kingdon (2003), the three streams—problems, policies, and politics—flow through the system independently until a policy window opens, causing them to converge and intersect [43].

The problem stream comprises issues recognized as public concerns requiring government action, thus entering the policymaking agenda [44]. The policy stream contains various solutions or programs developed by experts, which are evaluated to find a feasible solution [44, 45]. The political stream includes factors influencing political actors and the overall political climate, such as changes in executive leadership, shifts in public opinion, and lobbying by interest groups [44].

Drawing on Kingdon's model, the interaction among these three streams shapes the agenda-setting process. Effective policy implementation relies on their convergence, leading to the emergence of a policy window. Policy entrepreneurs play a crucial role in facilitating this convergence and creating opportunities for policy advancement [40]. Given the aim of this study to explore factors influencing the agenda-setting process for the pending medical tourism bill in Taiwan's FEPZs, this framework was deemed appropriate and utilized.

## Methods

Using Kingdon's multiple stream framework, this qualitative study explores the key factors shaping the problem stream of legitimizing medical tourism in the FEPZs, the proposed solutions in the policy stream for addressing medical tourism in the FEPZs, and the political events influencing the emergence of policies related to medical tourism on the agenda in the politics stream.

### Study design

Conducted as a qualitative case study, this research investigated the pending medical tourism provision of the FEPZs bill through extensive document reviews and semi-structured interviews with key informants.

### Document review

To establish a foundation on the medical tourism provision of the FEPZs bill, background information was collected through a comprehensive document review. This involved examining both peer-reviewed publications and grey literature, such as government reports, scientific literature, meeting minutes and newspaper articles, and encompassed the following steps. Initially, the PubMed database was searched to gather peer-reviewed publications on the topic of medical tourism in the FEPZs, employing keywords such as “medical tourism” or “health tourism “or “tourism, health” or “tourism, medical” and “free economic pilot zones” or “economic zone” or “special economic zone”. Secondly, searches were conducted on accessible ministry government websites, and minutes from meetings of the Executive Yuan, Taiwan, were manually searched and retrieved.

Additionally, the Google search engine was utilized for an additional search using "medical tourism in the FEPZs in Taiwan" as keywords to retrieve grey literature to ensure comprehensive coverage of any other relevant content. Moreover, the review was conducted in both English and Mandarin, as Taiwan's official language is Mandarin. The list of the analyzed governmental policy documents was summarized in Table 2.

**Table 2:** List of analyzed policy documents

No	Document	Publisher organization	Date of publication
1	FEPZs—Explanation of Medical Tourism Planning	MOHW	2013.03.27
2	FEPZs Planning Scheme (Approved Version)	Council For Economic Planning and Development (CEPD)	2013.04
3	Hearing on the Establishment of Specialized Medical Tourism Institutions in the FEPZs	MOHW	2013.07.10
4	First Stage Promotion Plan for the FEPZs	CEPD	2013.08.08
5	First Stage Promotion Plan for the FEPZs (Approved Version)	CEPD	2013.08.08
6	Impact Assessment Report on the Development of Medical Tourism Services and Trade Agreement	MOHW	2013.11.04
7	FEPZs- Medical Tourism Industry Related Tax Preferential Planning and Tax Expenditure Assessment Project Report	MOHW	2013.11.06
8	FEPZs Planning Plan (Amendment)	CEPD	2014.01
9	FEPZs-Medical Tourism (Health) Assessment Project Report	MOHW	2014.03.31
10	FEPZs Policy Description	National Development Council	2014.03.12
11	Draft Special Act for the FEPZs—Impact Assessment Report on the Opening of International Medical Institutions and its Impact on Taiwan's Medical and NHI System	MOHW	2014.03.20
12	"Draft Special Act for the FEPZs" 1st Public Hearing Report	Economics Committee of Legislative Yuan	2014.03.31
13	"Draft Special Act for the FEPZs" 2nd Public Hearing Report	Economics Committee of Legislative Yuan	2014.04.02
14	"Draft Special Act for the FEPZs" 3rd Public Hearing Report	Economics Committee of Legislative Yuan	2014.04.03
15	"Draft Special Act for the FEPZs" 5th Public Hearing Report	Economics Committee of Legislative Yuan	2014.04.14
16	FEPZs aim to promote the development of the medical tourism and health industry, without affecting the medical treatment rights and interests of local citizens	MOHW	2014.04.29

All documents are in Mandarin and the titles are translated by the authors

### Data collection through interviews

A purposeful sampling approach was employed to initially select key informants, followed by a snowball method where the first set of key informants provided additional names of resource persons, contributing to data saturation. Our intentional selection focused on four relevant categories: policymakers, healthcare providers, academics, and non-governmental organization (NGO) employees, all of whom played roles in the healthcare policy process. All informants were contacted through in-person visits, phone calls or emails, and upon their consent to participate, the interviewer (first author) recruited them for data collection.

The inquiries primarily centered on the interviewee's understanding of medical tourism in the FEPZs and their individual involvement or engagement with the policy. Written consent for recording the interviews was obtained in all instances. Interviews were held at the interviewees' workplace or nearest restaurant, and all interviews were conducted in Mandarin and recorded.

A total of 13 in-depth interviews were conducted with various stakeholders, with an average age of 57.08 years and an average of 27.08 years of health professional experience alongside 10.31 years of involvement in medical tourism. The participants comprised ten men and three women. The authors aimed for gender balance in the study, but due to the predominant occupancy of positions of interest by men during the study period, only 23% of the interviewees were female. Among them, five participants represented healthcare providers, three individuals were designated as policymakers, three individuals held positions in academia, and the remaining two interviewees represented NGOs.

Our aim was to encompass participants from different sectors, spanning from the highest echelons of policymaking to frontline healthcare providers involved in the operations of medical tourism. The interview sessions were conducted from March to August 2023. All interviews were carried out face-to-face and recorded with the participants' informed consent. Most interviews were conducted individually, without the presence of additional participants, unless the interviewee had a companion. The interviews varied in duration from 12 to 80 min, and hand-written notes were taken during each session. Transcripts were provided to participants for verification of accuracy where applicable. The interviewing process continued until data saturation, when duplicate data and no new information were found from the previous two interviews, was reached. A topic guide, developed based on Kingdon's multiple streams theory, was employed, and it underwent a pre-test in two interviews. Subsequent adjustments were made to the topic guide based on the analysis results. The following questions were posed:

1. Problem stream: What challenges still unresolved and unaddressed make medical tourism a problem in Taiwan? What prompted the government to start working on the legitimation of the medical tourism in the FEPZs? What, in your analysis, caused the failure or pending establishment of the medical tourism industry in the FEPZs?
2. Policy stream: What potential policies and solutions have been proposed, found, and/or adopted by stakeholders (hospitals or groups) to address the legitimation of medical tourism in the FEPZs?
3. Politics stream: What are the political factors and issues that can influence the effective adoption of medical tourism -related policies in Taiwan?

### Analytical approach

A qualitative data analysis was conducted using a computer-assisted tool (NVivo version 12 software). Firstly, all interviews were transcribed and uploaded into NVivo, while selected policy documents were highlighted within the same software. Secondly, all transcribed interviews and documents underwent thorough reading and re-reading for familiarization. Thirdly, the codes and nodes, as outlined in Table 3, were utilized to capture the interviewees' responses and information from policy documents. Finally, all the information was categorized under the themes of “problem”, policy” and “politics”, aligning with the streams of the Kingdon model utilized in this study.

**Table 3:** A summary of nodes under the three streams of the Kingdon framework

	Problem	Policy	Politics
Nodes	1. Medical and NHI system in Taiwan 2. Diplomatic political issues 3. Experiences and feedback from previous programs	1. Medical tourism provision of the FEPZs bill 2. Golden Decade National Vision 3. The operation method of medical facilities	1. Political parties 2. Public acceptance 3. Pressure from academia and NGOs

### Ethics

Ethical approval for the research was obtained from the ethics committee of National Yang Ming Chiao Tung University (IRB number: YM 111133E). Signed informed consents were gathered from participants before the interviews, and key informants retained the freedom to withdraw from the study at any point and for any reason. To ensure anonymity, quotes from participants were fully anonymized by excluding details about their position and profession.

## Results

A total of 13 participants, including policymakers, healthcare providers, academics and NGOs employees, consented to participate in this study through interviews, and the results are subdivided into problem stream, policy stream and political stream based on Kingdon’s framework, with relevant data from documents being presented.

### Problem stream

1. Low domestic healthcare spending and cost containment pressures from the NHI program
  Taiwan implemented the NHI program since 1995. Over the years, multiple stringent prospective payment methods, such as global budgets, have been adopted to contain medical care costs. Taiwan’s current health spending as a percentage of gross domestic product (GDP) was only 6.1% in 2022, and the annual growth rate was relatively lower than that of other Organisation for Economic Cooperation and Development countries [46]. Facing strong cost-containment pressures from the NHI program, medical care providers in Taiwan view medical tourism as a possible solution to their financial strains [47].
  *“The main reason for the emergence of medical tourism is that under the global budget policy of the NHI program, Taiwan's medical care industry faces a hard expenditure ceiling. The industry cannot be upgraded, and then health care professionals’ income cannot be increased because revenues of hospitals and clinics mostly come from the NHI.”* Participant 1.
2. Diplomatic political issues (cross-strait relations)
  The diplomatic relationship between Taiwan and the PRC is characterized by complex political dynamics and historical tensions, resulting in difficulties in issuing visas to certain nationalities. The FEPZs are intended to receive minimal government interference and are predominantly situated beyond customs boundaries. Therefore, medical services offered in the health institution within the FEPZs could be more accessible for patients of certain nationalities as they may not need a visa to enter the FEPZs.
  *“Those patients wouldn't need to enter our country's borders, which could alleviate issues related to Taiwan visa concerns. Our country’s diplomatic situation is rather unique, and we may not offer the same visa convenience to certain international individuals."* Participant 4.
3. Concerns about the commercialization of medical care and reduced access to care among Taiwanese due to proliferation of medical tourism
  Despite assurances from the MOHW that citizens’ access to medical treatment will not be affected [30], concerns persist regarding the commercialization of medical care. It is worth noting that fewer than 1% of physicians actually practice in the medical tourism field [48]. In the 5^th^ session of the 8^th^ Legislative Yuan in 2014, the MOHW reported that in 2013, there were 4293 hospitalized medical tourists, accounting for only one out of every 10,000 total hospitalizations in the country, and only three out of every 10,000 total outpatient visits [49]. Nevertheless, some public health academics argue that the promotion of medical tourism fosters the establishment of a commercial medical system, which they believe diminishes governmental control and social responsibilities [50].
  A leader from Taiwan Healthcare Reform Foundation, an NGO aiming to improve the healthcare quality and patient rights in Taiwan, expressed concerns about the shift in focus from patient care to profit-making. They emphasized that behind commercialization or corporatization lies the exploitation of labor rights [51].
4. The nascent development of medical tourism
  Medical advertising, language barriers and pricing are cited as obstacles to promoting medical tourism, which could potentially be addressed through policy relaxations in the FEPZs.
  Unlike in countries such as Thailand, South Korea, and Malaysia, where medical tourism can be legally advertised, medical advertisement is still strictly prohibited in Taiwan. For instance, the majority of healthcare providers in Thailand engage in travel marts, fairs, trade exhibitions, seminars, conferences, and advertise in travel magazines in various countries with government support [52]. South Korea allows medical advertisements in foreign languages, while Malaysia relaxed its traditionally strict prohibition on medical advertising in June 2005, although advertising information still requires approval from the Medicine Advertisements Boards [53, 54].
  *“Medical regulations (prohibiting advertising), especially why advertising is not allowed abroad, I think it's reasonable that it cannot advertise in Taiwan because we have an NHI system. However, why can’t Taiwanese hospitals advertise in Malaysia or Indonesia?”* Participant 7.
  Additionally, language barriers and pricing issues were also raised by the interviewees, which may limit the development of the medical tourism industry in Taiwan. 61% of respondents (8/13) mentioned language barriers during the interviews.
  *“Language could be a significant issue in Taiwan. While doctors or receptionists may speak English, there is still much room for improvement among the most basic level of nursing staff in terms of language proficiency.”* Participant 2.
  *“Some Myanma medical tourists actually want to come to Taiwan for advanced medical treatment, but they are afraid to do so. The biggest reason is their concern about the language issue because they heard that they have to speak Mandarin in Taiwan.”* Participant 11.
  Thailand, Singapore, and India are the leading countries in Asia for medical tourism, collectively capturing 90% of the market in 2011 [55]. Thailand's medical tourism revenue rose from $5.72 billion in 2016 to $9.87 billion in 2019, integrating tourism with healthcare through supportive policies and private hospitals. India's top hospitals, offering exclusive medical and travel packages, saw revenue nearly double from $4.4 billion to $8.15 billion in the same period. Singapore's revenue grew from $2.05 billion to $4.21 billion, driven by a cross-ministry committee. Malaysia achieved a 90% revenue growth, reaching $520 million in 2019 [56]. However, according to a study on Grey System Theory in the medical tourism industry, Taiwan has shown slower growth compared to its regional counterparts in the Asia-Pacific region. Forecasts predict that Taiwan's medical tourism revenue will grow slowly to NT$20.5 billion, with an expected 777 523 medical tourists by 2025. The study also suggests that Taiwan could benefit from enhancing its cost competitiveness and marketing strategies to capitalize on future growth opportunities [57].
  *“The pricing of medical tourism is not determined by market mechanisms but rather adjusted according to the multiplier within the NHI system. I believe this adjustment might harm the future development of medical tourism because when foreigners see such low prices, they might raise doubts about the quality.”* Participant 7.

### Policy stream

1. An annual franchise fee will be used to subsidize the NHI program, and hospitals within the FEPZs are not allowed to contract with the NHI program
  The financing issue of the NHI challenges the Taiwanese government, and medical tourism is seen as a possible solution [47]. Therefore, Article 52 of the FEPZs bill stated that hospitals within the FEPZs are not allowed to become NHI-contracted hospitals, and both foreign and domestic residents must bear the full cost themselves [30, 58]. More importantly, Article 53 of the FEPZs bill specifies that medical tourism institutions in FEPZs must submit an annual franchise fee to the central health and welfare authority [30, 59, 60].
  However, Cheng et al. (2014) stated that the proportion, purpose, and other matters related to the annual franchise fees are authorized to be determined by the MOHW, but there is a blank authorization issue in this legislation. Taiwan Medical Cooperation also argued that the lack of specific and detailed planning regarding the regulation of the annual franchise fees, which are stated as the surplus from medical tourism institutions, and their infusion into the NHI. While the franchise fees may help to supplement the funding of the NHI program, the specific amount or the model for the infusion, along with tax considerations, should be planned and explained beforehand [51].
2. Limited number of demonstration medical tourism sites and services allowed
  FEPZs integrate several health-related institutions, such as medical, biotech, pharmaceuticals, rehabilitation, and wellness [61]. Within the FEPZs, one or two demonstration sites for medical tourism can be established, including one hospital with up to 200 beds, offering services limited to health checkups, cosmetic medicine, and routine surgery [30]. To promote economic liberalization and internationalization, and to encourage foreign professional teams to collaborate with local medical institutions in the FEPZs, Article 49 of the original FEPZs bill in 2013 proposed to allow the "corporatization" of medical providers within the FEPZs, and relax some restrictions under the Medical Care Act, such as the provision that juridical persons, which include corporate care and medical corporations, cannot be members of medical care corporations and foreigners cannot serve as chairpersons [58, 62]. However, the provision allowing the corporatization of medical providers received strong opposition from academia and the public regarding fears and potential unintended adverse consequences of healthcare commercialization. Consequently, in 2014, the MOHW announced that hospital corporatization is strictly prohibited in the FEPZs [30].
  The capital contributions of members, the number of directors, and the proportion of medical personnel and foreigners serving as directors among the members of the medical care corporations mentioned in the preceding paragraph will be regulated by the central health and welfare authority and are not directly indicated in the FEPZs bill [29, 58].
  *“Corporatization, seeking profit—aren't corporations already eroding the country's funds enough? The government is stigmatized.”* Participant 9.
  *"I feel that hospitals (medical tourism) can be developed, but it may still require a complete set (corporatization) rather than developing within the existing non-profit environment. It absolutely cannot be developed in a fragmented manner. If you want to adhere to the NHI model, then there is no need to proceed. That mindset is completely different."* Participant 13.
  *"Will the corporatization of healthcare erode the current NHI coverage? Will these high-quality healthcare professionals be drawn to these more lucrative places? It's like traditional industries all shifting towards the electronics industry. So, in fact, this isn't too big of a problem. The government just needs a certain level of regulation."* Participant 7.
3. Allowing foreign medical personnel without local licensing to practice within the institution of FEPZs
  To avoid affecting residents’ rights to health and to encourage foreign professional teams to collaborate with local medical institutions in the FEPZs, the MOHW intended to allow recruitment of 20% of foreign healthcare professionals in the medical facilities in the FEPZs, excluding those from the PRC, Hong Kong and Macau [30, 63, 64].
  Therefore, Article 50 of the FEPZs bill states that medical tourism institutions may employ foreign medical personnel to practice within the institution. The number or ratio of such foreign medical personnel employed shall be announced by the central health and welfare authority and is not subject to the requirement of holding a specialized professional certificate in the country. The qualifications, conditions, abolition and other management matters shall be determined by the central health and welfare authority.
4. Imposing restrictions on medical tourism practices of domestic physicians
  Manpower maldistribution and the shifting of manpower from public services to medical tourists have occurred in both Thailand and India [24, 49, 65]. Although the MOHW emphasized that fewer than 1% of physicians practiced in the field of medical tourism in Taiwan [48], issues regarding the maldistribution of domestic human resources are still being considered.
  Article 51 clearly indicates that domestic doctors who are not registered in medical tourism institutions within the FEPZs are not allowed to perform medical services in these institutions. However, this restriction does not apply to emergency situations, consultations, support between medical institutions, invited visits, or with prior approval, and it does not exceed the time limit stipulated by the central health and welfare authority.
  The MOHW suggested that the number of physicians potentially affected should not exceed 100 and introduced stringent regulations on the hours of support provided by domestic medical staff within the FEPZs [66], although the specific time limit was not specified.
  On the other hand, challenges regarding the recruitment and retention of personnel in the FEPZs have been noticed.
  *“I don't think anyone would stay there for the long term, so it will be difficult to maintain. Without a consistent staff, it's difficult for medical tourism institutions to thrive in the FEPZs.”* Participant 13.
5. The emphasis on the essence of demonstration in the FEPZs
  In FEPZs, the emphasis on the essence of demonstration regarding the implementation of medical tourism is crucial.
  These zones serve as experimental grounds where innovative strategies and practices are showcased. The MOHW indicates that due to numerous questions surrounding the legitimation of medical tourism in the FEPZs, the government proposes starting with small-scale initiatives to assess their effectiveness. Without taking action, the outcomes will remain unknown. If the demonstration effect is achieved, it should be applied nationwide, and there is no need to promote it specifically in this region. However, if adverse effects arise in the FEPZs, subsequent policies can be adjusted accordingly [51].
  *"If the demonstration is successful and none of the initial concerns outlined in the regulations arise, perhaps the implementation will be extended nationwide in Taiwan."* Participant 10.
  *"I believe that cell therapy can be more liberalized (in the FEPZs). For domestic patients, government officials may fear moral responsibility if the results are not favorable. However, for foreign patients, there are fewer moral issues, so it may be worth considering opening up."* Participant 8.
6. Regulation and accreditation
  Hospitals within the FEPZs must still adhere to strict management under the Medical Care Act of Taiwan. Their operations and financial arrangements must fully comply with the regulations for medical care corporations, including oversight by accountants, reviews by the central administration's accountants and medical management experts, and periodic inspections. Regular hospital accreditation and evaluations are also required.
7. PRC investment is not allowed in the FEPZs
  In light of diplomatic and political issues between the PRC and Taiwan, as well as the relationship between the FEPZs bill and the Cross-Strait Service Trade Agreement (CSSTA). The MOHW emphasized that neither accepting hospitals established by the PRC in the FEPZs nor accepting physicians or medical personnel from PRC to practice in Taiwan [67].

### Politics stream

1. Supports from the government
  The two major political parties in Taiwan are the Kuomintang (KMT) and the Democratic Progressive Party (DPP). The FEPZs bill was proposed by the KMT, which was the ruling party at that time.
  The legitimation of medical tourism in the FEPZs was an important policy proposed by President Ma Ying-Jeou as part of the “Golden Decade National Vision”. It aimed to promote economic liberalization and internationalization across the country [51, 68].
  The premier stated that the FEPZs bill would be a priority, with deliberations expected to conclude by the end of July 2014 during legislative sittings in February 2014.
  The benefits of developing medical tourism in Taiwan include economic growth, industrialization of medical services, internationalization of medical services, and tourism promotion through medical services [69]. The establishment of the FEPZs was also expected to advance Taiwan's participation in both the Trans-Pacific Partnership and the Regional Comprehensive Economic Partnership, which are major trade agreements involving multiple countries, aimed at promoting economic integration, trade liberalization, and economic growth within their respective regions [70]. A leader from the Taiwan External Trade Development Council echoed that the legitimation of medical tourism in the FEPZs could be anticipated based on the government's support [51].
  *“At that time, President Ma Ying-Jeou aimed to promote CSSTA. Consequently, these (CSSTA and FEPZs bill) were bundled together, with many initially hoping for a forced passage of these measures. Hence, these cases were all packaged under the umbrella of CSSTA and were dismissed together.*” Participant 9.
2. Oppositions from opposition parties
  DPP, despite holding only 40 seats compared to the KMT 64 seats in the Legislative Yuan [71], strongly opposed the FEPZs bill. The DPP argued that the concept of medical tourism, as proposed during their era, aimed to promote reputable medical technology worldwide without involving corporatization or being located within an FEPZ. The Tainan City Government, under DPP leadership, argued that the KMT should immediately cease the legitimation of medical tourism in the FEPZs, deeming it ridiculous and destructive as it undermined democratic principles [72].
  The development of medical tourism is based on the relationship between industry and government [37]. However, participants noted that the opposition from the DPP was primarily political. They emphasized that if decision-makers failed to plan or communicate with stakeholders and instead opted for the easiest solutions, they would likely face significant resistance.
  *“(The reason for the failed medical tourism provision of the FEPZs bill) The first is the political issue, which has not been resolved.”* Participant 1.
  *“The problem with KMT governance lies in the lack of coordination among its various departments and its less cohesive nature compared to the DPP. While the bill's conceptualization might have been sound, its execution suffered from significant gaps. There was a lack of communication and an inability to navigate the political atmosphere effectively. Consequently, when it came to passing through the legislature, it failed. At that point, not only did this bill fail to pass, but none of them were likely to."* Participant 9.
3. Public concerns and critics from academia and NGOs
  The provision for medical tourism in the FEPZs bill faced significant challenges from the public, particularly concerning concerns about commercialization or corporatization. Medical care is considered a public good to which all residents should have equal and easy access at reasonable costs, and the potential impact on citizens’ health rights was heavily emphasized by NGOs and public health academics.
  *“If you were to ask the public about establishing medical tourism in the FEPZs, the majority would likely oppose it. They may feel their access to medical services would be exploited.”* Participant 10.
  *“Are they (the premier and the minister) simply pursuing the easiest plan to meet the supreme leader's goals? Opting for the simplest solutions often results in the greatest resistance. This is a problem with the design, which will drive public opinion towards the opposition camp.*” Participant 9.
  Furthermore, concerns were raised regarding the lack of specific and detailed planning regarding location, scale, quantity, and eligibility criteria. Representatives from Taiwan Medical Cooperation highlighted issues regarding the regulation of annual franchise fees, which are stated as surplus from medical tourism institutions and are to be infused into the NHI [51]. While beneficial given the current inadequacy of domestic NHI funds, the specific amount or model for infusion, along with tax considerations, should be planned and explained beforehand [51].
  *“At that time, the government proposed investing in the medical tourism industry in the FEPZs, but the details were not clearly explained. Who exactly would be involved? Who would take the lead? What would be the hospitals' contribution to their profits? None of these were addressed. Without these specifics, the plans didn't materialize, so there is no need to discuss the details further.”* Participant 13.
  Cheng et al. (2014) stated that the proportion, purpose and other matters related to the annual franchise fees should be authorized to be determined by the MOHW, but there is a blank authorization issue in this legislation.
  *“The biggest issue in legitimation of medical tourism in the FEPZs is the issue of positioning. When operating (a hospital) within the FEPZs, do you intend to operate for profit or as a non-profit organization, meaning whether it will be a profit-oriented entity or medical care corporate? This needs to be defined first. Furthermore, the source of funding, whether it comes from the government, a Build-Operate-Transfer arrangement, or another method, must also be clarified. Additionally, what should be clearly specified is the size or scale of the hospital in question.”* Participant 3.
4. Skepticism from medical care providers
  The financial issue and the recruitment of personnel have been highlighted by medical care providers. The lack of sufficient details in the medical tourism provision of the FEPZs bill raises concerns and questions about policy legitimation. Investors spent NTD$ 2-3 billion, but the policy might be unable to operate later, hence, there will be huge consequences.
  *“At that time, the legitimation of medical tourism in the FEPZs seems to be a one-step solution. The constant appeal was to build hospitals. Initially, if you wanted to build a hospital there, you had to start with a single hospital. In reality, we all know that the funding and manpower required to build hospitals are immense.”* Participant 4.
  *“When it comes to a special zone, it's not effective to just bring in a few doctors. Even if you get renowned physicians, it won't work without a team and specialized equipment. Just relocating a doctor and offering them double or triple the salary won't be effective.”* Participant 6.

## Discussion

The present study aimed to investigate the possible reasons contributing to the failure or pending status of the medical tourism provision of the FEPZs bill in Taiwan through Kingdon’s agenda-setting framework. The key findings indicated that the medical tourism provision of the FEPZs bill experienced only a partial coupling of Kingdon’s multiple streams—while its problems were recognized and the political will of the supreme leader was emphasized, a plausible policy solution, however, was still missing. Therefore, pressures and resistances from the public, elite, or informed stakeholders/opinion leaders, and political oppositions were noticed.

A feasible technique, an acceptable value, and a tolerable anticipation of future constraints are the main components of the policy solution [73]. However, consensus on the former two was never reached. The medical tourism provision of the FEPZs bill contains an excessive number and scope of blanket authorizations granted to rulemaking authorities, with four out of five articles of the bill being blank authorization clauses for the rulemaking authorities to establish related regulations independently. The lack of specific and detailed planning regarding location, scale, quantity, eligibility criteria, and especially the amount of annual franchise fees have been proposed by academics and representatives from NGOs [51]. Therefore, consensus on technical feasibility and value acceptability was difficult to obtain.

Moreover, strong opposition from national sentiment was noticed. As medical care is commonly perceived as a public good and equity in access to and quality of care are highly valued in Taiwan, concerns about social stratification, medical commercialization, or corporatization resulting from expanding medical tourism are major hindrances to the passage of the bill.

The role of party politics, particularly the tension between the KMT and the DPP, played a significant role in the legislative process of the FEPZs bill. During the legislative session in 2013–2014, the KMT held a majority in the Legislative Yuan (64 out of 113 seats), but the DPP, leveraging strong opposition, managed to stall the bill's progress. The opposition was not necessarily rooted in the merits of the bill but in the political dynamics between the parties. This political tension is crucial to understanding the legislative impasse. However, since the KMT was the ruling party with a majority in the legislature, party politics was a significant factor but not the sole reason for the bill's failure.

The study results echoed previous research showing that the Taiwanese government has ambitiously promoted the medical tourism provision of the FEPZs bill and has repeatedly emphasized the benefits it will bring to the country. However, there are still many concerns among the public, and the bill remains a subject of ongoing controversy, failing to reach consensus [74]. Our study found that technical feasibility and the lack of public acceptance were major barriers to further progress of the medical tourism provision of the FEPZs bill. This contrasts with economic benefits from previous thesis reports, which stated that when economic benefits were clearly lucrative, service providers showed higher acceptance of Taiwan's development of medical tourism services in the FEPZs, but cross-strait political risks were not perceived as a threat to them [31]. The difference between the current study and the previous one may stem from differences in research subjects. Previous studies only surveyed medical administrators, while the current study's subjects encompass policymakers, healthcare providers, academics, and NGO employees, among others.

Kingdon indicated that for issues to emerge on the agenda, they must meet a crucial precondition involving the coupling of problem, policy, and politics streams. Furthermore, once the window of opportunity presents itself, it only remains open for a limited duration [43, 73]. While the Executive Yuan proposed the FEPZs bill under strong support from the highest government level, the Legislative Yuan failed to reach issue consensus and lacked a policy entrepreneur who could coordinate efforts in the legislative bodies with the agenda. Politicized arguments questioning the benefits of the medical tourism provision of the FEPZs bill appeared to hold stronger sway.

Regarding the research framework, Béland and Howlett (2016) argued that Kingdon’s framework was developed to examine agenda processes in the United States (US) and is suited to the US only. However, the model has been utilized in several empirical studies in other countries, such as Canada, France, Kenya, and Burkina Faso [42, 75], and has been used to analyze failed policies previously, such as the multicultural and gender-fair curriculum rule in the US in 1988 [76], the educational policy in Argentina in 1993 [77], and the climate change policy in the US in 2010 [78]. Although, to the best of the author’s knowledge, no study has been conducted to investigate the policy of legitimation of medical tourism in a specific zone using the framework, the structure is well-suited for studying the medical tourism provision of the FEPZs bill.

A few limitations should be noted. First, some individuals, particularly current policymakers in the health sector, hesitated to participate. Consequently, despite the research team's efforts, the participants included did not fully represent the intended community. This limitation, coupled with its descriptive nature, diminished the extent to which the findings can be generalized. Secondly, consumers and patient advocacy groups were not included in the study, as their opinions were considered mixed and potentially lacking in representativeness. Thirdly, there were only 13 study participants, and the study reached data saturation; therefore, the homogeneity of the study participants may be criticized, but the participants involved had the intended backgrounds, including healthcare providers, policymakers, academia, and employees from non-governmental organizations. Furthermore, the utilization of a cross-sectional design in this study could potentially compromise the validity of the findings.

## Conclusion

The medical tourism provision of the FEPZs bill in Taiwan experienced only a partial coupling of Kingdon’s multiple streams—while the problem was recognized, and political will from the supreme leader was emphasized, a plausible policy solution, however, was absent. This study demonstrated the policy window seemed to be opened between 2013 to 2014 after the proposed “Golden Decade National Vision” by the president, but the uncoupling of the streams ultimately resulted in pending or failure of passing the FEPZs bill.

The failure of the medical tourism provision of the FEPZs bill appears to have been sealed from the beginning: it was hastily drafted and poorly defined, and overlooked crucial aspects necessary for effective communication and legitimation, thus struggling to garner the necessary support. The Taiwan example serves as a lesson for other policymakers that successful policies require careful consideration of problems, solutions, and political dynamics, the identification and resolution of obstacles at all levels, and vigorous implementation.

## Data Availability

The data will be available by request.

## Abbreviations

CEPD: Council For Economic Planning and Development
CSSTA: Cross-Strait Service Trade Agreement
DPP: Democratic Progressive Party
FEPZs: Free Economic Pilot Zones
GDP: Gross domestic product
KMT: Kuomintang
MOHW: Ministry of Health and Welfare
NGO: Non-governmental organization
NHI: National Health Insurance
PRC: People’s Republic of China
US: United States
